# DEVELOPMENT AND VALIDATION OF THE MINNESOTA AL REPORT CARD FOR RESIDENT QUALITY OF LIFE DOMAINS

**DOI:** 10.1093/geroni/igad104.2047

**Published:** 2023-12-21

**Authors:** Taylor Bucy, Tetyana Shippee, Nidhi Kohli

**Affiliations:** University of Minnesota School of Public Health, Minneapolis, Minnesota, United States; University of Minnesota, Minneapolis, Minnesota, United States; University of Minnesota, Minneapolis, Minnesota, United States

## Abstract

Assisted living (AL) describes long-term residential care settings that emphasize person-centered services and choice in a “home-like” environment. There is considerable variability in how AL is defined, what services are provided, and how quality is measured. Minnesota serves as a unique policy environment in which to study AL. In May 2019, Minnesota funded the development of an AL Report Card to measure and report on the quality of AL from resident and family perspectives, created in partnership with the University of Minnesota. Literature review, stakeholder feedback, and pilot testing were completed in 2020-21 and the survey was implemented statewide in 2021-22. Using 2021 AL resident quality of life (QoL) survey pilot data (N=1,392), the goal of this work was to assess the reliability and psychometric properties of the survey in measuring 7 QoL domains: food, staffing, environment, engagement, autonomy, culture, and security. The 7 domains were measured using 6, 10, 3, 6, 5, 3, and 6 items, respectively. We found acceptable internal consistency for four QoL domains: food, staff, security, and engagement (McDonald’s omega and Cronbach’s alpha ≥0.6). Confirmatory factor analysis was performed for each individual factor using diagonally weighted least squares for ordinal data. We found high interfactor correlations between the three domains with poor internal consistency (environment, culture, and autonomy). We collapsed these three domains into one overarching domain – climate – and found satisfactory data-model fit. Findings of stability and validity of QoL report card domains have important implications for adoption outside of Minnesota.

